# Short-Term Treatment with Diminazene Aceturate Ameliorates the Reduction in Kidney ACE2 Activity in Rats with Subtotal Nephrectomy

**DOI:** 10.1371/journal.pone.0118758

**Published:** 2015-03-18

**Authors:** Elena Velkoska, Sheila K. Patel, Karen Griggs, Raelene J. Pickering, Chris Tikellis, Louise M. Burrell

**Affiliations:** 1 Department of Medicine, The University of Melbourne, Austin Health, Heidelberg, Victoria, Australia; 2 Baker IDI Heart and Diabetes Institute, Melbourne, Australia; Max-Delbrück Center for Molecular Medicine (MDC), GERMANY

## Abstract

Angiotensin converting enzyme (ACE) 2 is an important modulator of the renin angiotensin system (RAS) through its role to degrade angiotensin (Ang) II. Depletion of kidney ACE2 occurs following kidney injury due to renal mass reduction and may contribute to progressive kidney disease. This study assessed the effect of diminazine aceturate (DIZE), which has been described as an ACE2 activator, on kidney ACE2 mRNA and activity in rats with kidney injury due to subtotal nephrectomy (STNx). Sprague Dawley rats were divided into Control groups or underwent STNx; rats then received vehicle or the DIZE (s.c. 15 mg/kg/day) for 2 weeks. STNx led to hypertension (P<0.01), kidney hypertrophy (P<0.001) and impaired kidney function (P<0.001) compared to Control rats. STNx was associated with increased kidney cortical ACE activity, and reduced ACE2 mRNA in the cortex (P<0.01), with reduced cortical and medullary ACE2 activity (P<0.05), and increased urinary ACE2 excretion (P<0.05) compared to Control rats. Urinary ACE2 activity correlated positively with urinary protein excretion (P<0.001), and negatively with creatinine clearance (P=0.04). In STNx rats, DIZE had no effect on blood pressure or kidney function, but was associated with reduced cortical ACE activity (P<0.01), increased cortical ACE2 mRNA (P<0.05) and increased cortical and medullary ACE2 activity (P<0.05). The precise *in vivo* mechanism of action of DIZE is not clear, and its effects to increase ACE2 activity may be secondary to an increase in ACE2 mRNA abundance. In *ex vivo* studies, DIZE did not increase ACE2 activity in either Control or STNx kidney cortical membranes. It is not yet known if chronic administration of DIZE has long-term benefits to slow the progression of kidney disease.

## Introduction

Kidney disease is increasing in prevalence and incidence, and is associated with considerable morbidity and mortality [1]. Over-activation of the renin angiotensin system (RAS) plays a major role in the progression of kidney disease, and blockade of the classic arm of the RAS is recommended as first line therapy [2]. Within the RAS, angiotensin converting enzyme (ACE) converts angiotensin (Ang) I into the vasoconstrictor, hypertrophic and fibrotic peptide, Ang II, which mediates its effects via the angiotensin type 1 receptor (AT1R). In the “alternate” arm of the RAS, ACE2 [3,4], counterbalances the effects of the classic RAS through degradation of Ang II, and generation of the antifibrotic and vasodilatory peptide, Ang 1–7 [5].

ACE2 is present in the normal kidney and is localized to the glomeruli, where it is expressed in podocytes and mesangial cells [6,7], to proximal tubules, and to the collecting ducts and vasa rectae in the medulla [8]. The importance of the level of ACE2 expression in kidney disease causality come from studies of ACE2 inhibition, which worsened glomerular injury in a mouse model of type 1 diabetes [9], presumably due to the removal of a degradative pathway for Ang II, and from ACE2 gene knockout mice (KO) with type 1 diabetes, where accelerated kidney injury was ameliorated by AT1R blockade [10]. Kidney disease secondary to subtotal nephrectomy (STNx) is associated with increased kidney ACE and Ang II [11–13], and, depletion of kidney ACE2 activity in both acute [8] and chronic [14] STNx. Depletion of kidney ACE2 occurs in other models of experimental kidney disease including 2-kidney, 1-clip hypertension, [15] ischemia reperfusion [16] and lipopolysaccharide induced renal injury [17]. Taken together the data suggests that imbalance in the tissue RAS with upregulation of the deleterious ACE/Ang II pathway and loss of the protective ACE2/Ang 1–7 pathway may predispose to the development and progression of kidney disease. This concept has led to strategies to replenish ACE2 or to activate ACE2 [18].

Recombinant human ACE2 has been shown to prevent Ang II induced kidney disease and tubulointerstitial fibrosis [19] and to slow the progression of diabetic nephropathy in the Akita mouse model of type 1 diabetes by reducing renal Ang II levels and increasing Ang 1–7 [20]. Three activators of ACE2 have been described including resorcinolnaphthalein, 1-[(2-dimethylamino) ethylamino]-4-(hydroxymethyl)-7-[(4-methylphenyl) sulfonyl oxy]-9H-xanthene-9-one (XNT), [21] and diminazine acetruate (DIZE; C_14_H_15_N_7_ · 2C_4_H_7_NO_3_), an anti-trypanosomal drug [22] that has been reported to have off-target effects to activate ACE2. [23]

*In vivo* studies report beneficial effects with XNT and/or DIZE in experimental rat models of arterial hypertension, pulmonary hypertension, myocardial infarction (MI), diabetic heart disease and hypertensive pregnancy [21,24–27]. For example, 4 week treatment with subcutaneous (s.c.) XNT reduced blood pressure, improved cardiac function and reversed cardiac and renal fibrosis in spontaneously hypertensive rats (SHR) [21], and 30 days of daily oral XNT ameliorated diabetes–induced cardiac dysfunction in rats [24]. With regard to DIZE, a 4 week s.c. infusion prevented the development of experimental pulmonary hypertension in rats [27] and improved cardiac remodelling in rats with MI. [26]

There are no studies of ACE2 activators in experimental kidney disease. We therefore examined the effect of 2 weeks treatment with DIZE on blood pressure, kidney function and kidney ACE and ACE2 activity in Control and STNx rats. In STNx rats, DIZE had no effect on blood pressure or kidney function but decreased cortical ACE activity, and ameliorated the reduction in kidney ACE2 activity. In *ex vivo* studies, DIZE had no effect to increase ACE2 activity in either Control or STNx kidney cortical membranes.

## Methods

### Experimental Protocol

Experimental procedures were performed in accordance with the National Health and Medical Research Council of Australia guidelines for animal experimentation and were approved by the Animal Ethics Committee, Austin Health (#A2010/03903). Female Sprague Dawley (SD) rats (body weight of 190–200g) were housed in a 12:12h light-dark cycle, with *ad libitum* food containing 0.4–0.6% NaCl (Norco) and water. STNx (n = 20) was performed as described previously [8,28,29], with a right nephrectomy, and ligation of all but one of the extra-renal branches of the left renal artery. STNx rats were randomly allocated to receive DIZE (2 weeks s.c. 15mg/kg/day, n = 10) via osmotic minipump (Model # 2002, Alzet, Cupertino, CA, USA), or to Vehicle (n = 10). The dose and mode of delivery of DIZE is the same as previously published studies [26,27]. Control rats received DIZE (2 weeks s.c. 15mg/kg/day, n = 8) or Vehicle (n = 10). On day 13, rats were housed in metabolic cages, and 24h water intake and urine volume measured, and a urine sample collected for the measurement of creatinine (Cr), sodium and ACE2.

On day 14, rats were anaesthetised with intraperitoneal (i.p.) sodium pentobarbitone (60 mg/kg/body weight), and systolic blood pressure was determined using a catheter inserted into the left carotid artery. Rats were then killed by a lethal dose of sodium pentobarbitone, and the remnant kidney was removed, weighed, snap frozen in isopentane and stored at -80°C for activity assays.

### Drugs

Sodium pentobarbitone was obtained from Boehringer Ingelheim, Artarmon, NSW, Australia), DIZE from Sigma-Aldrich Australia.

### Biochemical analysis

Urinary and plasma Cr and sodium were measured using an autoanalyser (Beckman Instruments, Palo Alta, CA, USA).

### Kidney ACE activity and kidney, urine and plasma ACE2 activity

Kidneys were dissected into cortex and medulla, and membrane preparations performed as described previously [8]. Kidney ACE activity was measured using an enzymatic assay as described previously [29]. Briefly, 100μg of membrane protein was incubated at 37°C with the ACE substrate hippuryl-His-Leu (1mM) in a total volume of 50μl in the presence and absence of EDTA (10μM) for 30 min. The rate of substrate cleavage was determined by comparison to a standard curve of the product His-Leu and expressed as nmole of substrate cleaved/mg of protein/hr.

Kidney, urine and plasma ACE2 activity was measured using an enzymatic assay as described previously [28]. Briefly, 100μg of membrane protein, 50μl of urine or 20μl of plasma was incubated in duplicate with an ACE2-specific quenched fluorescent substrate (QFS), (7-methoxycoumarin-4-yl)-acetyl-Ala-Pro-Lys (2, 4-dintirophenyl); Auspep, Parkville, Victoria, Australia), with or without 100μM EDTA [28]. The rate of substrate cleavage was determined by comparison to a standard curve of the free fluorophore, 4-amino-methoxycoumarin (MCA; Sigma, MO, USA). For kidney ACE2 activity, data is expressed as nmole of substrate cleaved/mg of protein/hr, for urinary ACE2 activity, the data was corrected for 24hr urine excretion and results are expressed as nmole of substrate/ml in 24hr, and plasma ACE2 is expressed as nmole of substrate/ml of plasma/hr.

### *Ex vivo* effect of DIZE on ACE2 activity in kidney cortex

Kidney cortex membranes from STNx (n = 4) and Control (n = 4) rats were incubated with varying concentrations of DIZE (0.1mM, 0.1μM and 0.1nM) or control. ACE2 activity was measured as described above and results expressed as nmole of substrate cleaved/mg of protein/hr after 90 minutes of incubation.

### Kidney cortex ACE2 mRNA

Gene expression of ACE2 in kidney cortex homogenates was determined by real time quantitative RT–PCR (reverse transcription–PCR) [30]. This was performed using the TaqMan system based on real-time detection of accumulated fluorescence (ABI Prism 7700; PerkinElmer) as described previously [30]. Gene expression was normalized to 18S mRNA and reported as ratios compared with the level of expression in Control rats, which were given an arbitrary value of 1.

### Kidney cortex ACE2 protein

Western blotting for ACE2 was performed as previously described [31]. Renal tissue from rat was minced, resuspended in buffer containing 10mM HEPES, 150mMNaCl, 1mM EGTA, 5mM MgCl2, and 0.02% NaN3, 5% Triton x100 pH 7.4 to which 0.5μg/mL pepstatin (Sigma, St Louis, Mo., USA), 0.25mg/mL leupeptin (Sigma, St Louis, Mo., USA), 0.1mg/mL benzamidine (Sigma, St Louis, Mo., USA) and 0.1mg/mL bacitracin (Sigma, St Louis USA) and homogenized at 13,000 rpm with the Ultra-Turrax (Janke and Kunkel IKA, Labortechnik, Germany) and centrifuged at 1000g at 4°C for 30 min. The resultant supernatant was harvested and stored in aliquots at -80°C.

Samples (100μg protein) were loaded and run on a 12% sodium dodecyl sulphate (SDS)-denaturing gel system and were trans-blotted onto nitrocellulose filters (Hybond P, Amersham-Pharmacia biotech, Buckinghamshire, UK) using a transfer tank at 100V for 60 minutes. At the end of the transfer the filters were blocked with 1% BSA overnight at 4°C with gentle rocking. The primary ACE2 antibody (rabbit polyclonal against ACE2 residues 489–508 donated by Millennium Pharmaceuticals, Cambridge, MA, USA) was diluted 1/2000 in 1% BSA/TBS and incubated for 2 hours at room temperature. Non-specific staining was tested with 1% non-immunized rabbit serum. Loading was standardised for the renal expression of β-actin (Abcam, Cambridge, MA, USA) concentration of 1/5000 in 1% BSA/TBS. The membrane (PVDF—FL, Millipore, Massachusetts, USA) was then washed thoroughly three times in wash solution (TBS/Tween). Positive bands were developed using the Western Blotting Analysis system (Amersham-Pharmacia Biotech, Buckinghamshire, UK), in which HRP-labelled secondary sheep anti-rabbit antibody (Chemicon, Temecula, Ca, USA) was diluted at 1/2000 and incubated for 1 hour at room temperature. Exposed Biomax film of bands representing ACE2 protein and β-actin were quantified on an Automated Imaging System (Imaging Research Inc., St Catherines, Ontario, Canada).

### Statistical Analysis

Data are presented as mean ± standard error of mean (SEM). P values were calculated using a two-way analysis of variance (ANOVA), followed by post hoc Bonferroni tests (GraphPad Prism 6). The Pearson correlation coefficient was determined for the associations between variables using the data from untreated Control and STNx rats. Two-tailed P-values <0.05 were considered significant.

## Results

### STNx and renal function

Table 1 shows the changes in physiological and biochemical parameters after STNx and the effect of DIZE treatment. Following STNx, rats had poor weight gain (P*<*0.001), elevated mean arterial pressure (P<0.01) and hypertrophy of the remnant kidney (P*<*0.001). Renal impairment was present with increased urinary protein (P<0.001) and reduced creatinine clearance (CrCl; P<0.001) compared with Control rats. STNx rats had increased water intake (P<0.01) and urine volume (P<0.001), and increased sodium excretion (P<0.001). In rats with STNx, DIZE had no effect on blood pressure, urine output or renal function. The only effect of DIZE in Control rats was to reduce urine output.

**Table 1:** End-organ weights, physiological and RAS parameters, and urine biochemistry.

	Control		Subtotal nephrectomy		
	Vehicle	DIZE	Vehicle	DIZE
		(15 mg/kg/day)		(15 mg/kg/day)
	(n = 10)	(n = 8)	(n = 10)	(n = 10)
Body weight (g)	239 ± 3	230 ± 3	207 ± 8***	205 ± 9
Mean arterial pressure (mmHg)	111 ± 5	106 ± 3	156 ± 11**	155 ± 4
**Renal Parameters**				
Left kidney weight (g)	0.86 ± 0.01	0.84 ± 0.01	1.01 ± 0.03***	1.00 ± 0.03
Left kidney/body weight (g/100g)	0.36 ± 0.01	0.37 ± 0.01	0.49 ± 0.02***	0.49 ± 0.02
Water intake (ml/100g/24hr)	11.5 ± 1.2	9.7 ± 0.9	24.2 ± 4.0**	20.1 ± 2.1
Urine output (ml/100g/24hr)	4.3 ± 0.4	2.7 ± 0.4 #	14.3 ± 2.1***	13.1 ± 2.3
Urinary protein (mg/100g/24hr)	41.8 ± 2.9	39.6 ± 6.1	61.2 ± 6.6***	59.6 ± 4.1
Plasma creatinine (μmol/L)	18 ± 1	17 ± 1	54 ± 7***	44 ± 4
Creatinine clearance (ml/min)	2.20 ± 0.25	2.03 ± 0.19	0.98 ± 0.14***	1.10 ± 0.11
Sodium excretion (mmol/24hr)	0.30 ± 0.03	0.25 ± 0.04	0.87 ± 0.14***	0.80 ± 0.08
**RAS Parameters**				
Plasma ACE2 activity (nmol/ml/hr)	5.7 ± 0.5	5.2 ± 0.3	6.9 ± 0.3*	6.7 ± 0.4
Cortical ACE2 mRNA (AU)	1.0 ± 0.2	1.5 ± 0.5	0.4 ± 0.1**	1.0 ± 0.3 #
Cortical ACE2 protein (AU)	1.0 ± 0.2	0.9 ± 0.1	0.5 ± 0.1	0.8 ± 0.1

Data expressed as mean±SEM. AU, arbitrary units;

*P<0.05

**P<0.01

***P<0.001 disease effect (Control vehicle vs. STNx Vehicle).

#P<0.05 treatment effect (Vehicle vs. DIZE)

### Kidney cortex and medulla ACE and ACE2 activity

**Fig 1:**
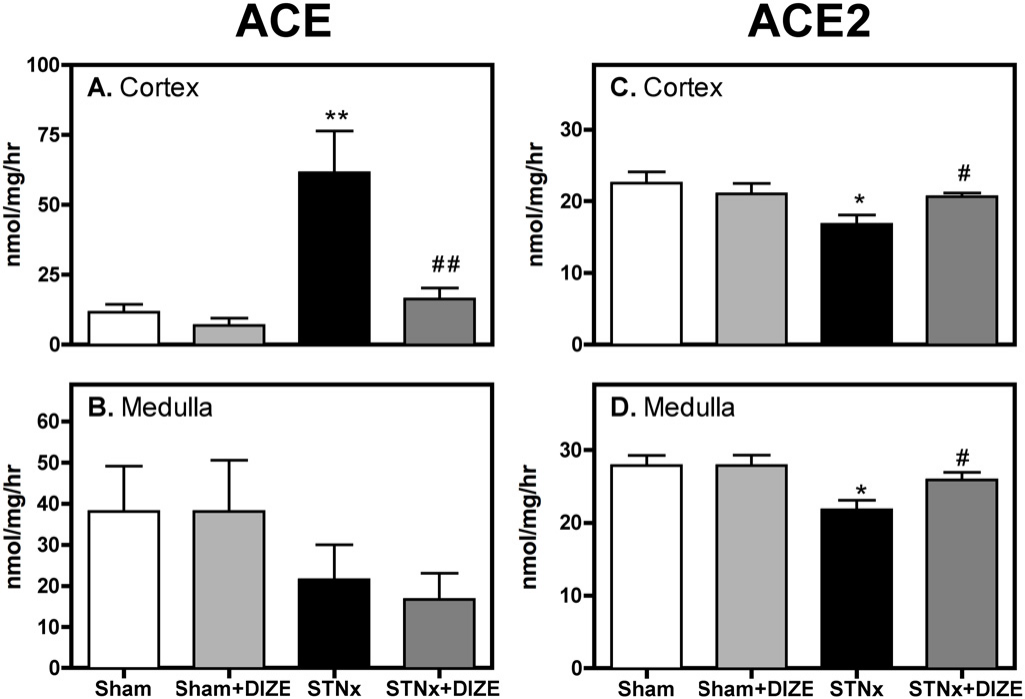
DIZE increases kidney ACE2 activity in subtotal nephrectomy rats. ACE and ACE2 activity in kidney cortex (A and C) and medulla (B and D) of Control (vehicle, n = 10; DIZE, n = 8) and STNx (vehicle, n = 10; DIZE n = 10) rats. Data expressed as mean±SEM. *P<0.05, **P<0.01, ***P<0.001 disease effect (Control vehicle vs. STNx Vehicle) and # P<0.05, ## P<0.01 treatment effect (Vehicle vs. DIZE)

ACE activity was 5-fold higher in the cortex of STNx compared to the Control cortex (Fig. 1A; P<0.01), but was unchanged in the medulla (Fig. 1B). DIZE treatment was associated with a significant reduction in ACE activity in the cortex (Fig. 1A; P<0.001) with no effect in the medulla (Fig. 1B). DIZE had no effect on kidney ACE activity in Control rats.

ACE2 activity was reduced in the cortex and medulla following STNx (Fig. 1C, D, P<0.05) and was increased in both regions following treatment with DIZE. DIZE had no effect on kidney ACE2 activity in Control animals.

We analysed the balance between ACE and ACE2 activity in the kidney. STNx was associated with an increase in the ACE/ACE2 activity ratio (0.55±0.14 vs. 3.85±0.77; P<0.01) that was reduced with DIZE (3.85±0.77 vs. 0.79±0.19; P<0.01), indicating a shift to a more favourable balance of the enzymes.

### Kidney cortex ACE2 mRNA and ACE2 protein

Cortical ACE2 mRNA was significantly reduced in STNx rats compared to Control rats (Table 1; P<0.01). DIZE treatment was associated with increased cortical ACE2 gene expression in STNx (P<0.05) but not in Control rats. Cortical ACE2 protein levels were not different between STNx and Control rats, and DIZE had no effect on ACE2 protein (Table 1).

### Plasma and urinary ACE2 activity

Plasma ACE2 activity was significantly increased in STNx compared to Control rats (Table 1; P<0.05) and did not change with DIZE treatment.

**Fig 2:**
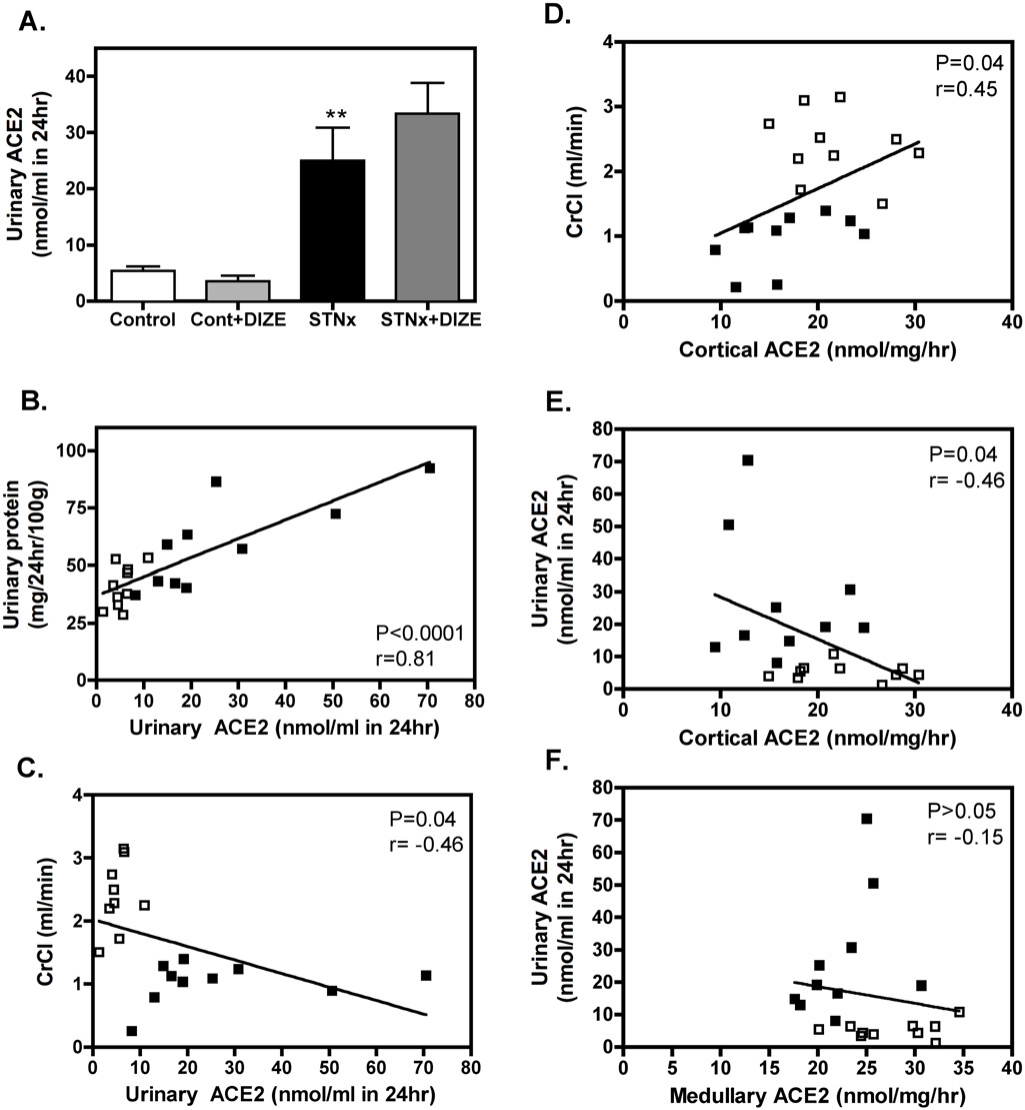
Increased urinary ACE2 activity is associated with decreased cortical ACE2 and impaired renal function. Urinary ACE2 activity (A) of Control (vehicle, n = 10; DIZE, n = 8) and STNx (vehicle, n = 10; DIZE n = 10) rats. Data expressed as mean±SEM. **P<0.01 disease effect (Control vehicle vs. STNx Vehicle). Increased urinary ACE2 activity was associated with increased urinary protein (B) and reduced creatinine clearance (CrCl; C), while cortical ACE2 activity decreased with impaired renal function (D). Increased urinary ACE2 activity was associated with reduced cortical ACE2 activity (E) but not medullary ACE2 activity (F). Only non-treated groups were used for correlation analysis (n = 20). Open squares represent Control rats; closed squares represent STNx rats.

Urinary ACE2 activity excretion was increased in STNx compared to Control rats (Fig. 2A; P<0.05) and unchanged with DIZE treatment. Increased urinary ACE2 with STNx was significantly correlated with increased urinary protein (Fig. 1B; P<0.001) and reduced CrCl (Fig. 2C; P = 0.04). Cortical ACE2 was also associated with impaired renal function with reduced cortical ACE2 correlating with reduced CrCl (Fig. 2D; P = 0.04). Furthermore, the reduction in cortical ACE2 activity (Fig. 2E; P = 0.04), but not medullary ACE2 (Fig. 2F), was associated with increased urinary ACE2, suggesting shedding of cortical ACE2 into the urine.

### *Ex vivo* effect of DIZE on ACE2 activity in kidney cortex

**Fig 3:**
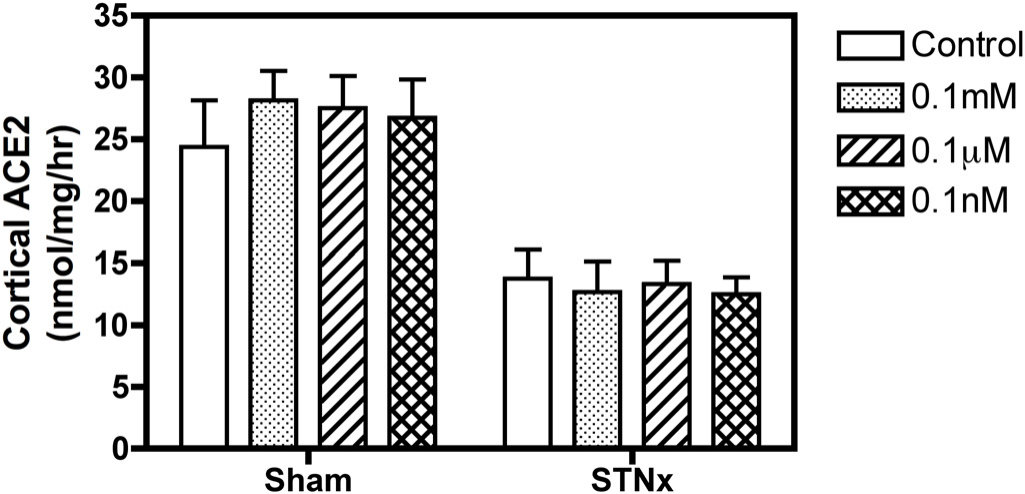
*Ex vivo* DIZE treatment had no effect on kidney ACE2 activity. Effect of DIZE (0.1mM, 0.1μM and 0.1nM) on endogenous ACE2 activity in kidney cortical membranes (100μg per well) from Control (n = 4) and STNx rats (n = 4). Data expressed as mean±SEM.

ACE2 activity was measured in the kidney cortex from Control and STNx rats in the absence and presence of DIZE at varying concentrations (0.1mM, 0.1μM and 0.1nM). As shown in Fig. 3, there was no effect of DIZE to increase ACE2 activity in either Control or STNx renal cortical membranes.

## Discussion

ACE2 is an important regulator of kidney function, but its role in kidney disease, and in particular that of non-diabetic origin has not been fully investigated. The results of this study confirm and extend our previous work that acute kidney injury with STNx leads to impaired renal function, increased cortical ACE activity and reduced ACE2 activity in the medulla and cortex [8,14]. In this 2 week study, s.c. infusion of DIZE, had no effect on blood pressure or kidney function in STNx rats, but was associated with a significant reduction in cortical ACE activity, and increased cortical ACE2 mRNA abundance and ACE2 activity. The *ex vivo* studies revealed that DIZE had no effect to increase ACE2 activity in either Control or STNx kidney cortical membranes.

The relative tissue balance of the deleterious ACE/Ang II pathway and the protective ACE2/Ang 1–7 pathway may be an important determinant of the *in vivo* effect of DIZE on ACE2 expression/activity. For example, DIZE had significant effects on ACE2 expression and activity in STNx rats with kidney RAS imbalance, but no effect on kidney ACE2 expression or activity in Control rats with a balanced RAS. MI is also associated with activation of the RAS with increased cardiac ACE activity and reduced ACE2 activity [26]. In rats with MI, 4 weeks of s.c DIZE improved cardiac remodelling in association with a significant increase in cardiac ACE2 mRNA expression and activity and a reduction in ACE mRNA expression and activity; these effects were negated by concurrent use of an ACE2 inhibitor, C-16 [26]. In rats with secondary pulmonary hypertension, characterized by a reduction in lung ACE2 activity, s.c. DIZE prevented the development of pulmonary hypertension in association with a significant increase in lung ACE2 activity [27]. Most recently, the effect of DIZE on the formation of Ang II-induced abdominal aortic aneurysms (AAA) was examined in male hypercholesterolemic (*Ldlr* ^-/-^) mice on either a wild-type or ACE2 deficient (*Ace*2^-/Y^) background [32]. Twenty-eight days of intramuscular DIZE (30mg/kg) led to significant increases in kidney ACE2 mRNA and ACE2 activity (measured by conversion of Ang II to Ang 1–7) in wild-type mice, and reduced the incidence and severity of Ang II-induced AAA. As DIZE did not produce any of these effects in ACE2-deficient mice, the results do support an ACE2-dependent mechanism of action for DIZE [32]. It remains unclear from the *in vivo* studies whether DIZE has direct effects to stimulate ACE2 activity, or whether the increase in ACE2 activity is secondary to its effects increase ACE2 mRNA abundance.

*In vitro* studies of DIZE on ACE2 activity have produced conflicting results [23,27,33,34]. The off target effects of DIZE to activate ACE2 were first reported by Kulemina *et al*. [8] who described that titration of ACE2 with DIZE (0.01–1000 μM) resulted in a biphasic dose–response curve; at low concentrations, the ACE2 was activated, whereas at high concentrations, it was partially inhibited. Shenoy *et al* [27] reported that incubation of human rACE2 with DIZE (100μM) led to increased enzymatic activity, whilst Haber *et al* [33] reported that neither XNT nor DIZE increased the enzymatic activity of mouse or human rACE2. Using mouse and rat kidney cortex lysates, Haber *et al* [33] also showed that neither XNT nor DIZE had a stimulatory effect on ACE2 activity, and that high concentrations of XNT and DIZE had an inhibitory effect on ACE2 activity. In the *ex vivo* experiments in the current study, we found no effect of DIZE to either increase or decrease ACE2 activity in kidney cortical membranes from Control or STNx rats.

In this study, as in studies in pulmonary hypertension, [27] DIZE treatment was associated with reduced tissue ACE activity, which is likely to be an indirect effect due to an improvement in tissue injury and therefore less ACE activation. This hypothesis is consistent with the finding that the degree of ACE “inhibition” with DIZE was much less than that observed with an ACE inhibitor such as ramipril, which causes almost 100% ACE inhibition [8] and the lack of effect of DIZE on blood pressure. [8,14]. In addition, *in vitro* studies have reported that DIZE had no effect on the catalytic activity of ACE [23].

We report for the first time that reduced cortical ACE2 activity was associated with increased urinary ACE2 activity levels, and that urinary ACE2 excretion correlated strongly with the degree of kidney disease as assessed by proteinuria and creatinine clearance. The source of ACE2 in the urine is thought to be the proximal tubules [35,36] and our finding that urinary ACE2 activity was negatively correlated with cortical ACE2 but not medullary ACE2 activity supports the tubular origin of urinary ACE2. Urinary ACE2 reflects cleavage of membrane-bound ACE2 by the proteinase ADAM17 (a disintegrin and metalloprotease) [37]. Previous studies have shown that inhibition of ADAM17 reduces renal fibrosis in angiotensin II-induced kidney disease in mice [38], suggesting that ADAM17 and its action to cleave ACE2 may play an important role in kidney disease. The intrarenal balance of the RAS components is critical in terms of disease progression, and loss of ACE2 from the tubules into urine may contribute to ongoing tissue injury and disease, through loss of a degradative pathway for Ang II and possibly decreases in Ang 1–7.

It is unknown if urinary ACE2 has potential as a biomarker of kidney damage in humans. Current reports focus on either ACE2 expression/activity in kidney biopsies or on ACE2 expression/activity in urine samples, with no studies to investigate both aspects. Tubulointerstitial ACE2 mRNA was decreased in patients with hypertensive nephrosclerosis and correlated with the degree of renal failure [39], and kidney ACE2 mRNA was reduced patients with type 2 diabetes and overt nephropathy [40]; neither study measured urinary ACE2 activity. It has been reported that urinary ACE2 was increased in patients with type 1 diabetes [41], type 2 diabetes [42], renal transplants [43], and chronic kidney disease [37], but these studies did not assess kidney ACE2 expression/activity.

## Conclusion

In summary, STNx rats have increased cortical ACE activity, and reduced cortical and medullary ACE2 activity with increased urinary ACE2 activity. Two weeks treatment with DIZE decreased cortical ACE activity and ameliorated the reduction in kidney ACE2 expression/activity, but had no effect to improve kidney function. The precise *in vivo* mechanism of action of DIZE is not clear, and its effects to increase ACE2 activity may be secondary to an increase in ACE2 mRNA abundance. Long-term studies with DIZE in kidney disease are warranted, ideally in combination with RAS blockade to assess if there is an incremental benefit of such a strategy to prevent progression to chronic kidney disease. Although DIZE is used for the treatment of trypanosomiasis or sleeping sickness, specific compounds that selectively amplify ACE2 activity will be needed, if such an approach is to be useful in the clinical context.
